# Mitochondrial Melatonin: Beneficial Effects in Protecting against Heart Failure

**DOI:** 10.3390/life14010088

**Published:** 2024-01-05

**Authors:** Russel J. Reiter, Ramaswamy Sharma, Luiz Gustavo de Almeida Chuffa, Fedor Simko, Alberto Dominguez-Rodriguez

**Affiliations:** 1 Department of Cell Systems and Anatomy, Long School of Medicine, UT Health San Antonio, San Antonio, TX 78229, USA; 2 Applied Biomedical Sciences, School of Osteopathic Medicine, University of the Incarnate Word, San Antonio, TX 78235, USA; 3 Department of Structural and Functional Biology-IBB/UNESP, Institute of Biosciences of Botucatu, Universidade Estadual Paulista, Botucatu 18618-689, São Paulo, Brazil; luiz_gustavo.chuffa@unesp.br; 4 Institute of Pathophysiology, Faculty of Medicine, Comenius University, 81108 Bratislava, Slovakia; fedor.simko@fmed.uniba.sk; 5 Servicio de Cardiología, Hospital Universitario de Canarias, 38010 Santa Cruz de Tenerife, Spain; adrvdg@hotmail.com

**Keywords:** arterial plaque, coronary artery, cardiovascular disease, cardiac fibrosis, diabetic cardiomyopathy, arterial plaque metabolism, hypertensive heart, melatonin actions, inflammation

## Abstract

Cardiovascular disease is the cause of physical infirmity and thousands of deaths annually. Typically, during heart failure, cardiomyocyte mitochondria falter in terms of energy production and metabolic processing. Additionally, inflammation and the accumulation of non-contractile fibrous tissue contribute to cardiac malfunction. Melatonin, an endogenously produced molecule, experimentally reduces the initiation and progression of atherosclerotic lesions, which are often the basis of coronary artery disease. The current review critically analyzes published data related to the experimental use of melatonin to forestall coronary artery pathologies. Collectively, these studies document melatonin’s anti-atherosclerotic actions in reducing LDL oxidation and triglyceride levels, lowering endothelial malfunction, limiting adhesion molecule formation, preventing macrophage polarization to the M1 pro-inflammatory phenotype, changing cellular metabolism, scavenging destructive reactive oxygen species, preventing the proliferation and invasion of arterial smooth muscle cells into the lesioned area, restricting the ingrowth of blood vessels from the vasa vasorum, and solidifying the plaque cap to reduce the chance of its rupture. Diabetic hyperglycemia, which aggravates atherosclerotic plaque formation, is also inhibited by melatonin supplementation in experimental animals. The potential value of non-toxic melatonin as a possible inhibitor of cardiac pathology in humans should be seriously considered by performing clinical trials using this multifunctional molecule.

## 1. Introduction

Cardiovascular disease (CVD) is a significant problem worldwide and creates a serious health and economic burden for both families and societies [1]. CVD, which is precipitated by many pathophysiological conditions, involves the malfunction of cardiomyocytes, especially at the mitochondrial level, when these organelles suffer in terms of the synthesis of ATP (energy production), calcium regulation, the accumulation of oxidatively-damaged debris, and the disordering of metabolic processing, among other cell biological disturbances. Moreover, inflammation is a contributor to CVD since it commonly leads to fibrosis. Fibrosis is a major component of the failing heart, which occurs when cardiomyocytes are replaced by non-contractile collagenous tissue, leading to diastolic and later systolic heart failure [2]. The inability of the remodeled heart to adequately force oxygenated blood throughout the circulation results in serious systemic problems, including edema, especially of the lower extremities, and, in more extreme cases, pulmonary hypertension and hepatic and renal damage may occur, as well as other cardiac disorders.

The most common contributor to CVD is coronary artery disease (CAD), which occurs when these vessels are narrowed as a result of the buildup of subendothelial fatty deposits, thereby depriving the cardiomyocytes of the optimal level of oxygen to sustain forceful contractions and sufficient relaxation [3]. The complete blockade of a coronary artery causes a heart attack, which is often associated with extensive cardiac damage and the replacement of cardiomyocytes with non-contractile fibrous tissue, thus weakening the ability of the ventricles to adequately move blood through the systemic circulation. Hypertension, especially when prolonged, damages the heart musculature by overworking the cardiomyocytes, which become weakened and rigid, thus inducing the same effect as damage inflicted by a heart attack in terms of the hypoperfusion of peripheral organs and right heart failure. Heart valves are essential for maintaining the optimal directional flow of blood. When diseased, the valves allow for the backflow of blood, causing stress on the heart and eventually weakening its contractility. Individuals with diabetes (T2D) have an elevated likelihood of developing coronary artery disease, in part due to the increased propensity of these patients to develop atherosclerotic plaques in their coronary arteries.

Heart failure is a complex clinical syndrome characterized by the inability of the heart to pump sufficient blood to the body due to structural and/or functional abnormalities. This is commonly indicated by a reduced cardiac output and/or an elevated intracardiac pressure at rest or during exercise. This syndrome affects more than 37.7 million people worldwide, and its prevalence is increasing [4]. This review evaluates the data that indicate that melatonin may be an effective treatment for slowing or halting atherosclerotic plaque formation and the progression of CVD.

## 2. Atherosclerosis and the Arterial Plaque

**Figure 1:**
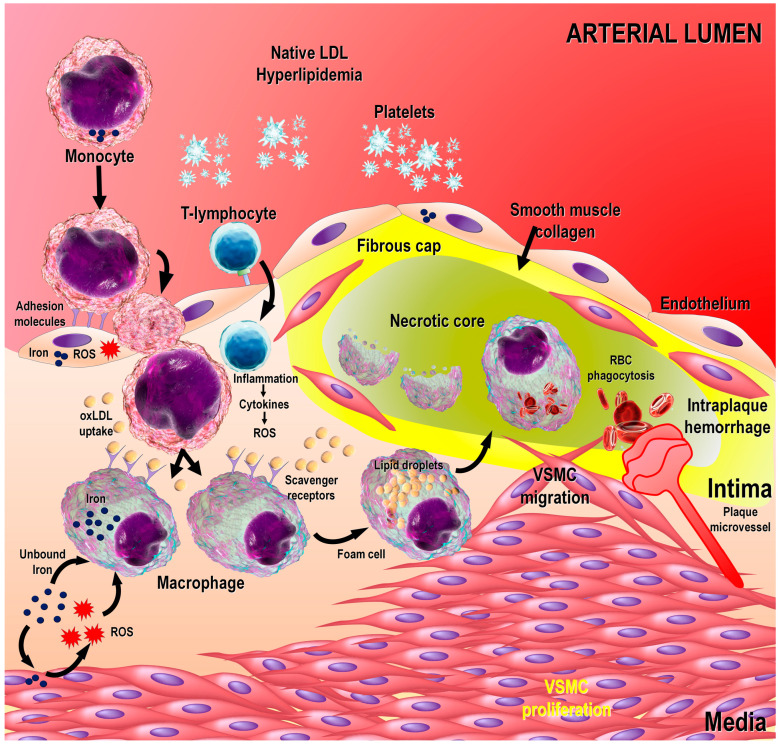
This is a simplified diagram of the arterial changes associated with the development of an atherosclerotic plaque and the potential points of intervention by melatonin. Endothelial malfunction plays a major role in the sequence of events, leading to the eventual maturation of an atherosclerotic plaque. The development of adhesion molecules (selectins, ICAM and VCAM, members of immunoglobulin superfamily) on the endothelial cells cause myeloid cells, e.g., monocytes and T-lymphocytes, to roll on and eventually stick to the endothelium. Monocytes undergo diapedesis and enter the subendothelial space and arterial intima. These cells are quickly transformed to phagocytic macrophages, which are polarized to the pro-inflammatory phenotype. Concurrently, oxidized low-density lipid (oxLDL) is taken into the subendothelial space and phagocytized by macrophages to form foam cells, which eventually degenerate to form the necrotic core. T-lymphocytes also enter the subendothelial space and contribute to the substantial inflammatory response that occurs. These inflammatory cells release cytokines, which generate free radicals (reactive oxygen species (ROS) and reactive nitrogen species (RNS)) which further contribute to tissue injury. Vascular smooth muscle cells (VSMC) in the arterial media proliferate and migrate into the atheroma. Along with collagen and other processes, e.g., calcium deposition, they form the fibrotic cap of the atherosclerotic lesion. The nature of the cap is important in the subsequent cardiovascular damage that may occur. Rupture of the plaque in a coronary artery can lead to a blockade of the distal blood flow, causing a heart attack; also, the plaque can become so thick that the vessel is occluded, and the downstream tissue becomes ischemic. The ingrowth of blood vessels from the vasa vasorum of the arterial media and externa further contributes to the failing plaque structure. Melatonin has multiple actions by which it resists atheroma formation and malfunction: (1) melatonin lowers circulating triglycerides and reduces the oxidation LDL cholesterol; (2) melatonin inhibits the development of adhesion molecules on the damaged endothelial cells; (3) melatonin impedes the polarization of anti-inflammatory M2 macrophages to the pro-inflammatory M1 phenotype; (4) melatonin scavenges the generated ROS to reduce molecular damage; (5) melatonin inhibits the proliferation and migration of the VSMC; (6) it has anti-angiogenic properties that resist the ingrowth of blood vessels from the vasa vasorum; (7) it stabilizes the atherosclerotic cap, which prevents its rupture, etc.

Generalized atherosclerosis, which is invariably accompanied by CVD, is a highly common condition in both well-developed and underdeveloped countries. It occurs in individuals of any ethnic background and the elderly are more often and increasingly afflicted with this dangerous condition [5]. Atherosclerosis involves a highly complex series of events, which include endothelial dysfunction, subendothelial lipid accumulation and foam cell formation, and vascular smooth muscle cell elaboration. [6] (Figure 1). Many risk factors have been identified that predispose an individual to this menacing disease; some of the most serious of these are genetic factors, hypertension, smoking and sustained hyperglycemia. One pathophysiological manifestation of this disease includes the formation of atherosclerotic plaques in the arterial vascular wall, which contribute to its serious nature [7]. Plaques, made up primarily of oxidized low-density lipoprotein cholesterol, so-called “bad” cholesterol, and miscellaneous cells which secrete destructive cytokines, narrow the small arteries of the heart such that the downstream cardiomyocytes are starved of oxygen, weakening their ability to carry out the usual workload. CAD is commonly associated with atherosclerosis of the entire systemic arterial vasculature [8,9]. CAD causes chest pain or discomfort known as angina; in many cases, it is a prelude to a heart infarction.

## 3. Altered Plaque Metabolism

When the normal endothelium is damaged, monocytes undergo diapedesis between the endothelial cells to enter the intima, where they are transformed into macrophages and phagocytize the lipoproteins, resulting in the formation of foam cells [10] (Figure 1).

Endothelial cells may display glycolysis under normal conditions but when the process becomes exaggerated, it aids atheroma formation by interfering with endothelial cell barrier function and advancing pathological neoangiogenesis, which alters the vulnerability of the plaque for rupture. Excessive aerobic glycolysis, followed by the increased acidification of the microenvironment, promotes the polarization of M2 to M1 macrophages, which exaggerates inflammation in the plaque. Moreover, vascular smooth muscle cells (VSMC) proliferate around the plaque, contributing to the collapse of the arterial lumen. As with other cells in the plaque, VSMC take advantage of aerobic glycolysis and proliferate excessively [11,12].

A chronically elevated inflammatory response is a highly menacing aspect of an arterial lesion. This is driven by innate myeloid cells and eventually involves both innate and adaptive immune responses [13]. The greater the immune response, the more intense the aerobic glycolysis of the associated cells; thus, it becomes a vicious cycle which results in the further stimulation of plaque formation, rendering it less stable [14]. Also, the formation of the proinflammatory phenotype of the monocyte/macrophage is a result of the metabolic shift from oxidative phosphorylation (OXPHOS) to aerobic glycolysis with an elevated uptake of glucose and an accelerated glycolytic flux, which leads to the generation of the proinflammatory cytokines IL-1β and IL-6 [15].

**Figure 2:**
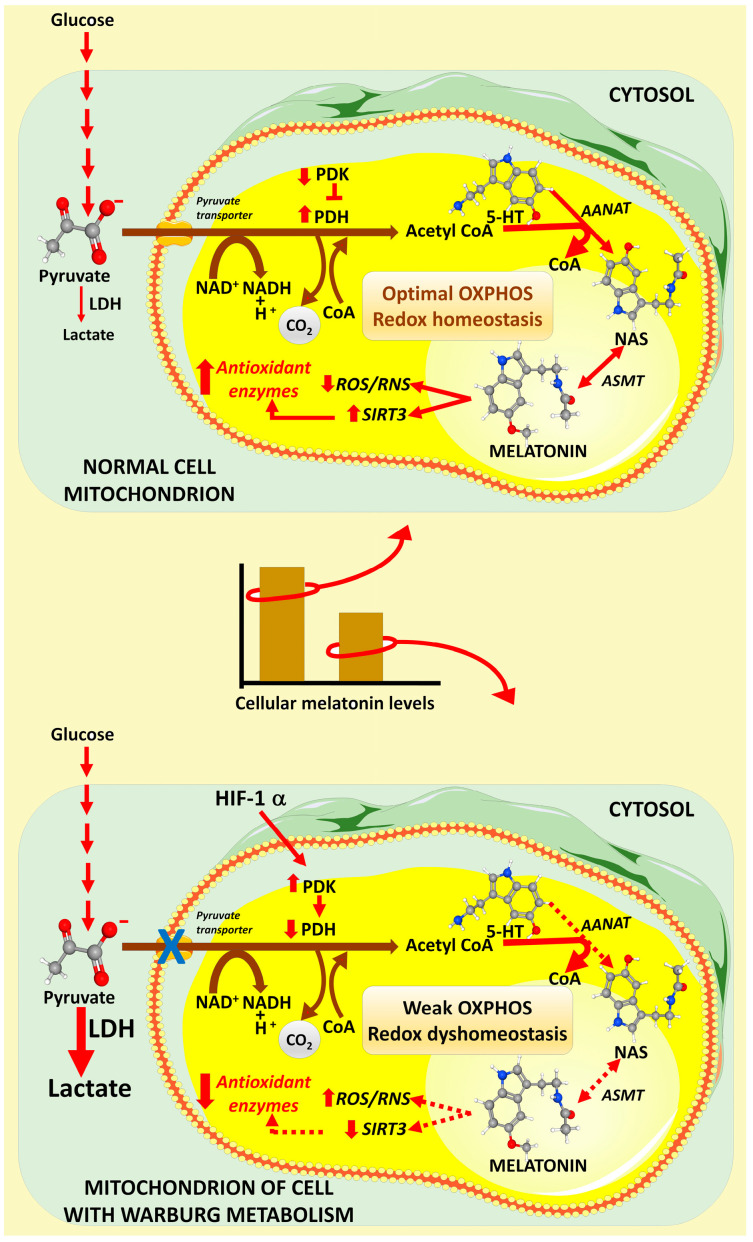
In the mitochondria of non-diseased cells (**top** panel), pyruvate, the end-product of glucose metabolism, is transported into the mitochondria via the pyruvate transporter. In the mitochondria, pyruvate is converted to acetyl coenzyme A under the influence of pyruvate dehydrogenase complex (PDH). Acetyl coenzyme A plays an important role in enhancing the activity of the citric acid cycle, which, in turn, supports optimal oxidative phosphorylation. Additionally, acetyl coenzyme A is a necessary cofactor/cosubstrate for acylalkyamine-N-acetylransferase (AANAT) for the conversion of serotonin (5-HT) to N-acetylserotonin (NAS), the immediate precursor of melatonin. NAS is metabolized to melatonin by acetyl serotonin methyltransferase (ASMT). In the mitochondria, melatonin, as it does elsewhere, functions as an efficient reactive oxygen (ROS) and reactive nitrogen species (RNS) neutralizer while also upregulating sirtuin 3, which promotes antioxidative enzyme activities. This combination of effects aids in the maintenance of mitochondrial redox homeostasis and ensures unaltered oxidative phosphorylation and ATP production. Preliminary evidence shows that normal cells manifesting adequate acetyl coenzyme production have higher melatonin concentrations than pathological cells (**middle** panel). Many diseased cells, such as those in an arterial plaque (**bottom** panel) convert to Warburg-type metabolism, which interferes with the intramitochondrial transfer of pyruvate; this results from the downregulation of PDH by the activated pyruvate dehydrogenase kinase (PDK), which, in turn, is stimulated by hypoxia-inducible factor 1α (HIF-1α). In the cytosol, pyruvate is converted to lactate, which is released from the cell and acidifies the microenvironment of the cell, which also promotes its pathological progression. The relative deficiency of acetyl coenzyme A, as occurs in Warburg-type metabolism, lowers the intramitochondrial production of melatonin (**middle** panel) leading to redox dyshomeostasis and weakens oxidative phosphorylation and ATP production. These changes contribute to cardiovascular disease and heart failure. Up arrows indicate stimulation; down arrows indicate inhibition.

Lactate, the end-product of anaerobic glycolysis, is formed intracellularly when pyruvate is metabolized to lactate by lactate dehydrogenase (LDH). In normal cells, pyruvate enters the mitochondria via the pyruvate transporter, where it is irreversibly converted to acetyl-coenzyme A (AcCoA) by pyruvate dehydrogenase (PDH). AcCoA feeds the citric acid cycle, supports OXPHOS, and serves as a cofactor for mitochondrial melatonin synthesis [16] (Figure 2). During glycolysis, the upregulation of pyruvate dehydrogenase kinase (PDK) inhibits PDH, thereby restricting the entrance of pyruvate into the mitochondria and shunting it into the fermentation pathway [17] to generate lactate. Lactate is then transported out of the cell via the monocarboxylate transporter (MCT), of which there are numerous subtypes [18]. Additionally, MCT transporters in endothelial cell membranes cause the uptake of lactate into these cells, which inactivates prolyl hydroxylase and stabilizes hypoxia-inducible factor-1α (HIF-1α); this promotes the ingrowth of blood vessels from the vasa vasorum in the adventitia and media of the artery into the plaque, making them more likely to rupture due to weakening of the cap [19,20]. Moreover, the additional blood vessels provide extra nutrients and oxygen, which support plaque progression and increase the likelihood of intraplaque hemorrhage [21]. Many factors contribute to plaque neovascularization, plaque instability, and plaque rupture, e.g., necrotic core expansion, the secretion of adipokines and cytokines from cells of the arterial wall, and augmented inflammation [22].

In the event of any of the emergency scenarios mentioned above, where free radical-mediated cardiomyocyte damage occurs due to hypoxia resulting from coronary artery occlusion, re-establishing a well-oxygenated blood flow by stent placement or drug treatment (thrombolytics, blood thinners, nitroglycerin, etc.) multiplies injury to the downstream cardiac cells, especially at the level of the mitochondria [23]. This damage, which is generally known as reperfusion injury, is also believed to be a consequence of elevated free radical generation in the re-perfused tissue [24]. Independent of the cause of the interrupted blood supply to an area of the heart, since cardiomyocytes do not proliferate, any cells that die are replaced by tissues that are incapable of contracting, so the movement of blood from the heart to the systemic circulation is impeded [25]. This has numerous negative systemic consequences and leads to heart failure (HF), a frequent cause of death in post-cardiac-arrest patients [26].

## 4. Melatonin and Arterial Plaque Integrity

Arterial plaques are classified relative to their propensity to rupture, i.e., either as a soft plaque which is prone to rupture or as a stable hard plaque which is less likely to do so. When a plaque does rupture, the necrotic debris that escapes can clog the artery downstream and cause a vascular embolism. These secondary consequences, rather than the presence of a plaque per se, are the usual major causes of death in individuals with atherosclerosis [27]. Soft plaques contain cholesterol, fatty compounds and fibrin, a blood clotting material. Hard plaques are often calcified or fibrous, especially at the surface, with the softer material underlying this crust; hard plaques are much less likely to rupture and to cause a heart attack, so the conversion from soft to hard plaques is beneficial in terms of preventing cardiac ischemia/hypoxia [28].

After developing a model of rupture-prone vulnerable plaques in a hypercholesterolemic ApoE^−/−^ mouse, we found that the oral administration of melatonin for nine weeks reduced plaque rupture and the subsequent formation of intraluminal thrombus [19,28]. Mechanistically, melatonin markedly limited inflammation in the plaque by converting M1 macrophages to the M2 phenotype, thereby stabilizing the plaque; this change related to the AMPKα/STAT pathway and involved the RORα nuclear melatonin receptor. Also using an ApoE^−/−^ mouse model of atherosclerotic plaques, Li et al. (2019) reported that melatonin enhanced their stability by stimulating prolyl-4-hydroxylase α1 (P4Hα1) [29]. This is a crucial enzyme involved in intracellular collagen maturation and subsequent secretion, and melatonin solidified the plaques and presumably made them less likely to rupture. This information is especially relevant to individuals who already have documented atherosclerotic deposits in their coronary arteries or elsewhere. It is also highly relevant to the aged, where endogenous melatonin production is generally diminished, thereby increasing the propensity for plaque rupture.

A plaque does not have to rupture to cause a cardiac disorder; when one becomes so large that it interrupts blood flow to a portion of the heart, it renders the downstream cardiomyocytes hypoxic, causing them to die and leading to their eventual replacement by cells/tissues that are of little value to the pulsating heart. Cardiomyocytes are especially sensitive to even short intervals of hypoxia, since they are extremely rich in mitochondria (up to 35% of the cardiomyocyte cell mass may be comprised of mitochondria), all of which rely on adequate oxygen to mediate OXPHOS and a high level of ATP production to meet the extreme energy demands for contraction and relaxation.

The obstruction of a coronary artery can happen quickly when, in response to a change in shear stress, lymphocytes adhere to the surface of the endothelial cells and, along with platelets, accumulate at the site and occlude the vessel. The endothelial cells become “sticky” when they express members of a family of immunoglobulin-like adhesion molecules that includes ICAM-1, ICAM-2, VCAM-1 and PECAM-1; this also aids in the transendothelial migration of the lymphocytes [30]. Melatonin has long been known to inhibit adhesion molecule expression on endothelial cells, which diminishes the likelihood of the occlusion of a coronary artery due to the buildup of lymphocytes [31,32] (Figure 1). Experimental evidence documents that this action of melatonin is mediated by the nuclear receptor for melatonin (RORα)/signal transducer and activator of transcription 3 (miR-223-STAT-3) signaling pathway [33]. Additionally, melatonin increases the levels of endothelial nitric oxide (NO), which causes endothelium-dependent relaxation and inhibits the aggregation of platelets at the lesion site, thereby also increasing the flow of blood through the artery [34]. Melatonin, via its membrane receptor, also decreased vascular calcification by attenuating osteogenic differentiation and the senescence of VSMC via transfer of exosomes containing miR-204/miR-211 in a paracrine manner. Importantly, the internalization of VSMC exosomes into mouse artery contributed to a reduction in vascular calcification [35].

The rupture of blood vessels within a plaque can also cause a rapidly expanding hematoma, which exaggerates the narrowing of a coronary artery lumen, leading to an acute interruption of the blood supply to the downstream tissues, thus inducing a locus of ischemia. Moreover, the vessel walls in the vicinity of an atherosclerotic plaque are weakened by the lesion; as a result, aneurysms develop and contribute to the death of patients with advanced atherosclerosis [5].

## 5. Melatonin’s Protective Actions against CVD and Cardiac Damage

**Figure 3:**
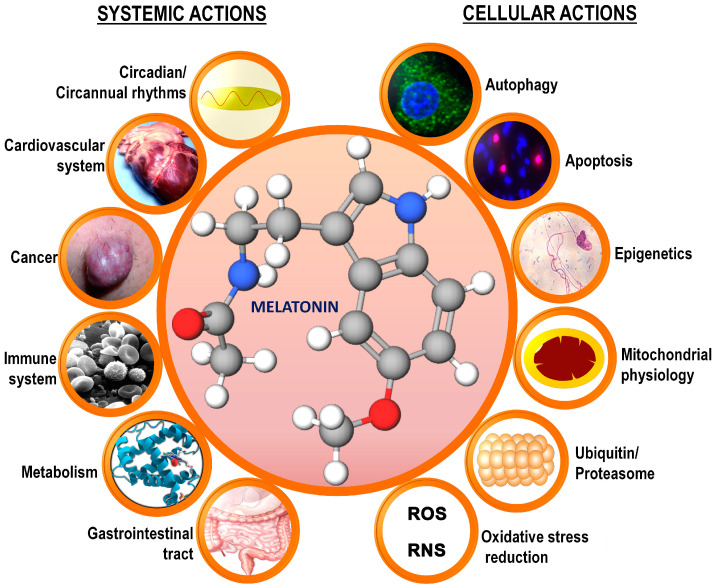
A summary of some of the multiple systemic and cellular actions of melatonin in mammals, including the human; many of these functions are interrelated with cardiovascular disease. The circadian rhythm of melatonin influences virtually every system in the body, with disturbances in this rhythm contributing to alterations in cardio-vascular physiology. Inflammation is a major aspect of arterial plaque formation, with melatonin functioning as an anti-inflammatory agent. The metabolism of pathophysiological cells at the level of the mitochondria is frequently changed when cells adopt a Warburg-type metabolism; this occurs in an arterial plaque when M2 anti-inflammatory macrophages are polarized to the M1 pro-inflammatory phenotype. The conversion to Warburg-type metabolism also results in a change in the mitochondrial acetyl coenzyme A synthesis. This agent normally feeds the citric acid cycle and supports oxidative phosphorylation. Acetyl coenzyme A is also a cofactor/cosubstrate for the conversion of serotonin to N-acetylserotonin, the precursor of melatonin, in the mitochondria, so the changes in the availability of this cofactor likely reduce the synthesis of mitochondrial melatonin. Melatonin is a potent direct reactive oxygen species (ROS) and reactive nitrogen species (RNS) scavenger, and also less directly removes these reactants from the mitochondria by upregulating sirtuin 3, which, in turn, stimulates superoxide dismutase 2. The reduction in melatonin in arterial plaques likely contributes to plaque formation and cardiovascular disease, as summarized in this report.

Melatonin is a multifaceted, endogenously produced molecule that functions in the scavenging of ROS/RNS, influences apoptosis, has a major effect on mitochondrial physiology, and seems to positively influence every system in the body (Figure 3). With regard to atherosclerosis, melatonin interferes with this process using numerous means. While the early findings particularly recognized the ability of melatonin to reduce atherosclerotic degradation due to its antioxidant and anti-inflammatory actions, more recent studies prove that melatonin’s protective actions are far more expansive than originally envisaged [36]. For example, it reduces the oxidation of LDL cholesterol, a major component of coronary artery plaque, such that it impedes plaque formation (Figure 1) [37]. Melatonin lowers circulating triglycerides, which are also involved in fat deposit buildups in the arterial intima [38]. Moreover, melatonin decreases hyperglycemia by augmenting glucose uptake by normal tissues, which reduces the hyperglycemia increment and thereby limits the events that spur atherosclerosis [39,40]. Finally, accumulated evidence indicates that melatonin reduces hypertension, which is a contributory factor to endothelial damage and atheroma formation [41,42,43,44].

Relative to melatonin’s antioxidative actions and inflammation-inhibitory effects, these actions clearly modulate atherosclerotic plaque initiation and development. While hyperglycemia associated with diabetes is highly detrimental to the vascular endothelium because of the marked degree of oxidative stress that it causes, melatonin has vasculoprotective actions in these cells due to its extensive free radical scavenging properties [45,46,47]. Since melatonin shelters the coronary artery endothelium from hyperglycemia-mediated oxidative damage, it would be expected to defer the progress of an atheromatous abrasion. Moreover, melatonin diminishes the inflammatory component of the plaque by preventing the polarization of the anti-inflammatory M2 macrophage to the proinflammatory M1 phenotype [48,49] (Figure 1). The polarization of macrophages between the M1 and M2 varieties relates to the metabolism of L-arginine, with the phenotypes differing in whether they convert arginine into inducible nitric oxide synthase (iNOS; common in the M1 macrophage) or primarily into urea (common in M2 inflammatory macrophages) [50]. Melatonin switches the metabolic fate of L-arginine from urea to NO in macrophages, thereby maintaining them as M2 anti-inflammatory phenotypic cells.

**Figure 4:**
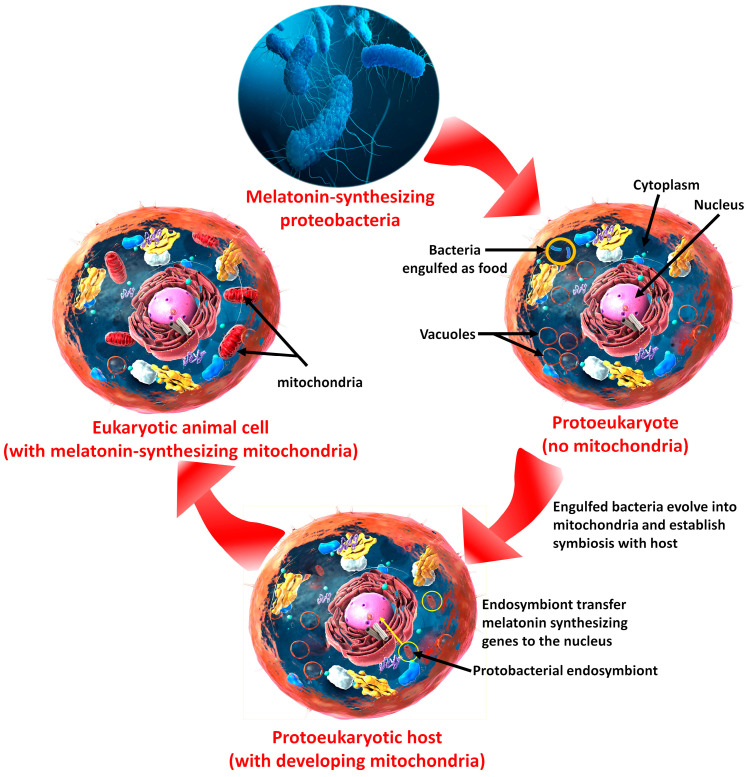
This figure illustrates how melatonin presumably became associated with the mitochondria of all eukaryotic cells. Early in their evolution (about 2.5 billion years ago) protokaryotes, the precursors of present-day true eukaryotic cells engulfed melatonin-synthesizing proteobacteria as food. Eventually the ingested bacteria established a mutually beneficial symbiotic association with their host cells (known as the endosymbiotic theory for the origin of mitochondria); this was a major asset to the developing eukaryotes because the transformed bacteria (in the form of mitochondria) provided the host cells with an important energy source via OXPHOS, which the proteo-bacteria already had. Since OXPHOS is a high free-radical-generating process and the proteobacteria presumably also had the ability to synthesize the efficient antioxidant, melatonin, this molecule was also conserved (see Figure 2). Throughout their evolution, both OXPHOS and melatonin synthesis were theoretically preserved such that all present-day eukaryotic cells possess both processes.

Melatonin’s ability to neutralize ROS/RNS and stimulate antioxidative enzymes that metabolize them to innocuous molecules unquestionably plays a central role in the ability of melatonin to reduce endothelial damage and thereby postpone the inception of an atheroma [36,51]. However, when an atheroma continues fatty substance buildup and forms a plaque, it may rupture. As already discussed, when this occurs, the plaque debris may eventually interrupt the blood flow to a portion of the heart, resulting in hypoxia and glucose deprivation in the blood-deprived cells; hypoxia is a major stimulus for free radical generation, which is highly destructive to cellular and molecular physiology, especially at the mitochondrial level [24,52]. Thus, neutralizing free radicals (ROS/RNS) in hypoxic tissue is highly effective in minimizing some of the ensuing molecular damage. Melatonin, in addition to directly scavenging free radicals, has multiple other functions for suppressing the devastating actions of toxic oxygen derivatives; as a result, it has a long investigative history of reducing ischemic/hypoxic damage in numerous tissues, including in the heart [53]. The scientific literature is replete with publications documenting the ability of melatonin to forestall damage resulting from hypoxia/reoxygenation injury in multiple organs [54,55,56]. Its efficacy as an inhibitor of oxidative damage in any tissue including cardiomyocytes is, at least in part, likely related to the high reported concentration of melatonin in the mitochondria, which are also the site of abundant ROS/RNS generation [57]. The high levels of melatonin in these organelles are an evolutionary feature resulting from eukaryotes engulfing melatonin-synthesizing bacteria, along with their subsequent transformation into mitochondria (Figure 2 and Figure 4) [58]. Considering the seriousness of ischemic/reperfusion lesions, it is not surprising that melatonin has been extensively experimentally and clinically tested to overcome the damaging actions of oxygen/glucose deprivation at the level of cardiomyocytes [59,60,61].

A detailed analysis of the mechanisms whereby melatonin interferes with inflammation as it relates to atherosclerotic plaque development was recently provided [62]. A variety of methodologies were used to examine atherosclerotic lesion development in ApoE^-/-^ mice fed a high-fat diet (HFD). They identified S100a9 as a critical mediator of the protective actions of melatonin in deferring atherogenesis. In HFD-fed mice, S100a9 was markedly overexpressed at both the mRNA and protein levels; this caused a pronounced activation of NF-ĸB signaling and upregulated vascular inflammation. In HFD-fed mice given melatonin during the same 12-week period, the S100a9/NF-ĸB pathway was significantly suppressed, leading to a reduced vascular inflammatory response and a slower atheroma enlargement. The authors concluded that, in consideration of their findings related to the ability of melatonin to attenuate vascular inflammation, the regular use of melatonin may be worthy of consideration as an adjunct therapy to resist atherogenesis.

Throughout this report, the data documenting the ability of melatonin to efficiently reduce the levels of tissue damage resulting from excessive ROS/RNS generation was emphasized. There is no way of knowing, however, what percentage of melatonin’s protection was due to its radical scavenging ability compared to the ancillary means by which this molecule functions as an antioxidant, i.e., the stimulation of antioxidant enzymes, inhibition of pro-oxidant enzymes, metal chelation, and other processes. Likewise, while the protective actions are usually attributed to melatonin, its metabolites, which are a result of its scavenging processes, are, in some cases, better scavengers that melatonin itself. Thus, there is a series of melatonin metabolites that are likely involved in the scavenging of toxic oxygen-based reactants; this is referred to as the antioxidant cascade [63]. Some of the metabolites of melatonin involved in the cascade include cyclic 3-hydroxymelatonin, N-acetyl-N-formyl-5-methoxykynuramine, N-acetyl-5-methoxykynuramine, and perhaps others [64,65].

## 6. Melatonin and Cardiac Fibrosis

Fibrosis is common after a tissue is damaged with this process, leading to the replacement of the injured cells with connective tissue; the treatment options for these conditions are severely limited. Substituting fibrous connective tissue, which is primarily collagen type 1, for damaged cardiomyocytes, although meant to be a reparative process, is not compatible with an optimally functioning heart since these fibers are non-contractile and therefore weaken the contractile force of the heart and reduce the ejection fraction. The fibrogenic response by myofibroblasts occurs in four phases, all of which are negatively impacted by melatonin. Indeed, melatonin has been documented to have anti-fibrotic actions in multiple organs, initiated by many different factors; when it occurs in the heart, it has clear pathological consequences and is a major contributor to HF.

The first evidence indicating that melatonin is essential in limiting fibrosis development in the heart was the accumulation of cardiac collagen in pinealectomized-melatonin-deficient rats [66]. Myocardial fibrosis also invariably follows injury to the heart and is initiated by inflammation and necrosis of the injured cardiomyocytes. Melatonin has significant anti-inflammatory and antioxidative actions, which suppress its anti-fibrotic activity [67]. When fibrous tissue accumulates in the wall of the left ventricle, the organ compensates by adding additional cells and collagen, leading to left ventricular hypertrophy/remodeling (LVH), which is partially suppressed by supplemental melatonin [68]. In the heart, the excess collagen and glycosaminoglycans (GAG) are contributions of myofibroblasts, which are driven by cytokines such as TGF-β1 and PC1; these agents are known to be downregulated by melatonin [69]. Light at night, which suppresses the nocturnal rise in melatonin synthesis and its levels in the circulation, worsens cardiac remodeling after experimental myocardial infarction. The elevated damage seems to be a result of stimulation of the brain–heart sympathetic nervous system axis, which exaggerates the size of the infarct area with the development of fibrosis. Supplementation with melatonin overcame much of the cardiac injury by reversing cardiac remodeling, probably due to the suppression of the brain–heart sympathetic hyperactivation [70]. In a rat model of metabolic syndrome, orally administered melatonin beneficially modulated the electrical activity of the heart (normalizing the QT interval) as well as the left ventricular expression of KCNK and KVNH2 genes, both of which encode cardiac potassium ion channels [71]. The potential clinical applications of the use of melatonin to correct pathologies associated with cardiac remodeling due to an infarct or to melatonin syndrome have recently been reviewed [72]. The evidence is that the causes of heart failure could be diminished if melatonin was used during the critical period of cardiac repair. There is also preliminary clinical evidence that treatment with intravenous melatonin in patients with acute myocardial infarction meant that patients went without hospital admission for heart failure, on average, 90 days longer than those treated with placebo [73].

## 7. Hypertensive Heart and Melatonin

Heart failure (HF) is a syndrome induced by a broad spectrum of cardiovascular disorders. Hypertension and ischemic heart disease are the principal pathologies underlying about 70% of the cases of heart failure development. The hypertensive heart with left ventricular hypertrophy (LVH) is frequently damaged by chronically increased blood pressure [74]. LVH is a compensatory reaction to increased muscular stress when the hemodynamic overload is distributed to greater numbers of muscular mass units, thereby reducing wall tension and ATP consumption. With a prolonged duration of overload, the structure and function of the left ventricle deteriorate. The development of LVH is a biological strategy to reduce the transition to HF [75,76,77]. Thus, the nature of the regressed heart and its prognostic impact depend not only on the type, severity, or duration of LVH, but also on therapeutic means of inducing LVH degradation. Among the principal factors improving the prognosis is the reduction in the excessive fibrotic rebuilding of the hypertensive heart and improvements in the energy state and electric stability [75,77,78]. Based on data that were predominantly derived from animal studies, melatonin seems to be an effective means of hypertensive heart protection [79].

There is evidence that melatonin may participate in myocardial fibrosis development. In continuous light-induced hypertension with limited melatonin production, fibrotic rebuilding was observed in terms of enhanced insoluble and total collagen levels, along with enhanced advanced oxidation protein products in the LV and aorta. The administration of melatonin in this model reduced the oxidative load in target tissues and prevented the enhancement of hydroxyproline concentration in the insoluble collagen fraction and the total hydroxyproline in the LV [78].

In an L-NAME-induced NO-deficient model of hypertension, melatonin reduced the collagen content and concentration rise in the LV without influencing the reduced NOS activity [80]; it also prevented the increase in soluble and total collagen concentration in the LV without an effect on the renin-angiotensin-aldosterone system. Of note, however, angiogenesis II, and its downstream products were not increased but decreased in L-NAME-induced hypertension [43]. In a lactacystin-induced hypertension model, melatonin reduced the concentration of collagen in the LV in association with elevated LV nitric oxide synthase activity [81]. In a regression experiment using spontaneously hypertensive rats (SHR), melatonin depressed the level of hydroxyproline in the insoluble collagen protein in the LV [82]. Likewise, in a similar study using L-NAME-induced hypertension, melatonin improved the restoration of endothelium-derived constricting factor signaling, reduced the oxidative load in the aorta, and restored femoral artery remodeling [83]. Interestingly, in neither of these experiments did melatonin elevate LV mass, potentially because the principal factor of heart muscle growth, the elevated blood pressure (BP), was only slightly (although significantly) reduced by melatonin. Moreover, melatonin attenuated angiotensin II-induced cardiomyocyte hypertrophy in cell culture via the CypA/CD147 signaling pathway, reducing ROS production [84], and, in Ang-IIinduced hypertension in mice, melatonin attenuated cardiac hypertrophy and improved mitochondrial function via mitochondrial calcium uptake 1 (MICU1) [85].

In a clinical situation, it was shown that an abundantly expressed proinflammatory cytokine that stimulated oxidative stress, cyclophilin A, was negatively correlated with melatonin in patients with LVH, and a low melatonin level was considered a pathogenetic factor in LVH development [86]. Even more importantly, reduced melatonin levels were associated with the development of heart failure in patients with hypertensive cardiomyopathy, and the level of melatonin was suggested to be a potential predictor of heart failure in the hypertensive heart population [41].

## 8. Melatonin and Diabetic Cardiomyopathy

**Figure 5:**
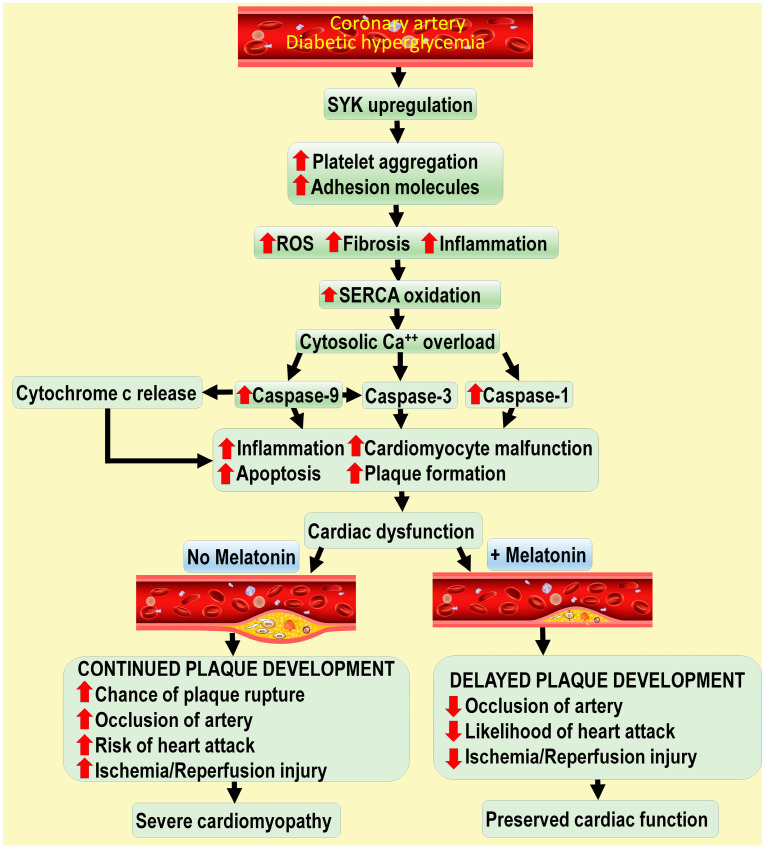
Some of the mechanisms by which diabetic hyperglycemia aggravates atherosclerotic development are outlined in this figure. Hyperglycemia induces a pronounced upregulation of spleen tyrosine kinase (SYK). This is followed by a series of processes that enhance endothelial malfunction and inflammation, with a substantial rise in damaging reactive oxygen species (ROS) production; the oxygen-based reactants contribute to the oxidation of sarcoplasmic reticulum transport ATPase (SERCA), which leads to an elevation in cytosolic calcium, thereby stimulating caspases (cysteine–aspartic proteases) that are involved in advancing inflammation and promoting cell-killing (apoptosis), cardiomyocyte failure and the stimulation of plaque formation. These pathologies compromise global cardiac function and further contribute to cardiovascular disease. As indicated in the lower right of the illustration, supplemental melatonin deters the processes of atherosclerotic progression and plaque formation. Additional details related to plaque formation and melatonin protection are summarized in Figure 1. Up and down red arrows mean stimulation and inhibition respectively.

Cardiomyopathy commonly accompanies type 2 diabetes (T2D). Based on the known actions of melatonin, Zhou and colleagues predicted that melatonin would limit the development of diabetic cardiomyopathy induced by elevated glucose [79]. In a rodent model, they observed that high glucose levels and the progression of atherosclerosis were associated with elevated cardiac spleen tyrosine kinase (SYK) activity. SYK is a multifunctional cytosolic enzyme involved in a variety of biological activities, some of which clearly relate to atherosclerotic damage to the vascular endothelium, i.e., platelet activation, adhesion molecule upregulation, and innate immune function. [87] (Figure 5). When high-blood-glucose animals were given supplemental melatonin or if SYK was genetically ablated, diabetic cardiac malfunction was ameliorated, cardiac fibrosis was reduced, and there was a significant preservation of cardiomyocyte viability. The identified mechanisms documented that activated SYK suppressed the activity of mitochondrial complex 1, causing an overproduction of ROS. The excessive reactive oxygen derivatives led to the oxidation of sarcoplasmic reticulum transport ATPase (SERCA), such that cellular calcium could not be sequestered in the sarcoplasmic reticulum (SR), causing cytosolic calcium (Ca^2+^) overload [88]. Elevated cytosolic calcium levels subsequently activated caspase 9 and caspase 12, leading to both mitochondrial and SR damage, which contributed to cardiomyopathy [89,90]. The results show that defects in the SYK/COX-1/SERCA pathway, as they relate to calcium signaling, clearly aggravate diabetic cardiomyopathy, with the response being abrogated by melatonin (Figure 5).

The observations of Zhou and colleagues in reference to melatonin’s ability to rescue the heart from damage resulting from diabetic hyperglycemia have been repeatedly confirmed using the measurement of different endpoints [79]. Zhang et al. noted that, with the aid of echocardiography, daily melatonin administration reduced left ventricle remodeling and preserved cardiac physiology [91]. Mechanistically, the benefits of melatonin stem from its ability to depress the apoptosis of cardiomyocytes, increase autophagy and preserve mitochondrial function. According to Xiong and colleagues, the efficacy of melatonin in reducing cardiomyocyte endoplasmic reticulum stress-mediated apoptosis was also a means by which the indoleamine protected against diabetic cardiomyopathy [92]. Moreover, a major aspect of the melatonin’s ability to shelter cardiomyocytes from elevated glucose levels during diabetes likely involves preservation of mitochondrial function [93,94]. The collective findings indicate that melatonin’s protection of the heart from deterioration under diabetic cardiomyopathic conditions is unequivocal and multifunctional [95,96,97]. Considering the serious nature of cardiovascular and cardiac damage in individuals suffering with T2D, melatonin supplementation could be considered a reasonable and likely effective means of combating this seriously destructive condition (Figure 5).

Melatonin also attenuates cardiac collagen buildup in mice that are hyperglycemic due to chemical destruction of the endocrine pancreas [98]. In this thorough in vivo/in vitro study of diabetic cardiomyopathy, the workers showed that melatonin both improved cardiac physiology and lowered heart collagen content in mice with diabetes; the protective actions of melatonin involved the inhibition of the TGF-β1/SMAD signaling pathway and inactivation of the NLRP3 inflammasome. Similar beneficial actions of melatonin were observed when cardiac fibroblasts were exposed to a high glucose medium. Che and co-workers also found the knockdown of miR-141 in cultured fibroblasts negated the antifibrotic actions of melatonin [98]. Because of their comprehensive findings, they urged clinical investigations into the possible utility of melatonin as an agent to protect the heart from diabetic hyperglycemia.

## 9. Molecular Mechanisms of Melatonin’s Protective Actions

The role of the innate immune system in supporting a sustained proinflammatory response in atherosclerotic plaques is well-established; this inflammatory response is mediated in large part by the monocyte/macrophage system, which is a prominent feature of an atherosclerotic lesion [99] (Figure 1). Given that galectin-3 stimulates lipoprotein uptake into subendothelial foam cells and enhances inflammation-induced endothelial damage, and that it is involved in the regulation of the immune system and inflammatory responses [100], Wang and colleagues tested whether galectin-3 expression in atherosclerotic lesions would be impacted by melatonin [101]. In both in vitro and in vivo studies in a mouse model, they reported that melatonin effectively reduced galectin-3, followed by the downregulation of NF-ĸB and enhancement of transcription factor EB (TYEB) nuclear localization. These changes were accompanied by a stimulation of autophagy, a process that protects against vascular inflammation, since atherosclerotic macrophages are normally functionally impaired [102,103]. In light of their findings, Wang and co-workers suggested that melatonin may have clinical applications in patients with myocarditis because of its ability to downregulate galectin-3 [101].

As in every other cell, mitochondrial dysfunction in cardiomyocytes has a critical role in contributing to disease (Figure 2). Altered mitochondrial physiology is especially relevant to the heart musculature since cardiomyocytes have an unusually high concentration of mitochondria commensurate with the heart’s requirement for large amounts of energy, i.e., ATP. Additionally, the mitochondria also have many other responsibilities in protein assembly, signal transduction, apoptosis regulation, etc. Fluctuations in mitochondrial Ca^2+^ levels perturb muscle contraction coupling along with the stimulation of elevated ROS, especially during periods of ischemia and reperfusion. The augmented ROS cause further damage to proteins of the respiratory chain and they also contribute to mitochondrial membrane damage, depressed ATP production, augmented Ca^2+^ levels, and the opening of the mitochondrial permeability transition pore (mPTP), allowing for the escape of cytochrome *c,* which eventually leads to apoptosis [104,105]. Collectively, these changes contribute to endothelial malfunction, to plaque buildup and to plaque instability, all of which worsen the likely outcome of atherosclerotic lesions.

In addition to and concurrent with its ability to neutralize toxic oxygen derivatives in the mitochondria [106,107] (Figure 2), melatonin also inhibits NOS, maintains calcium homeostasis, and stimulates mitochondrial sirtuin 3 (SIRT3) [108,109]. Additionally, the disturbed molecular processes caused by mitochondrial calcium overload all seem to be ameliorated by melatonin [36,110].

Interestingly, subjects with measurable atherosclerosis have an attenuated nocturnal rise in melatonin, a natural modulatory agent [111]. Moreover, atherosclerosis is usually most prominent in aged individuals, a time at which endogenous melatonin levels are usually markedly diminished. These findings should prompt an epidemiological investigation into the degree of melatonin suppression and the severity of atherosclerosis and cardiovascular disease.

## 10. Human Studies

A host of different approaches have examined whether melatonin influences the destructive outcome of myocardial infarction (MI) in humans, associated with atherosclerotic lesions of the coronary arteries. Endogenous plasma levels of melatonin have also been compared in individuals suffering from acute myocardial infarction (AMI), those with dilated cardiomyopathy (DCM), and in control subjects. The patients in both diseased categories were found to have lower circulating endogenous melatonin values than those measured in the control subjects, with the depressed levels correlating with the severity of myocardial damage and cardiac output, especially in the DCM subjects [112]. A recent finding links sleep deprivation or reduced melatonin levels with CVD at the level of endothelial inflammation and atherogenesis via circulating exosomes in distant communications [113]. In this study, exosomes extracted from the plasma of volunteers with or without sleep deprivation were evaluated to determine changes in miRNA expression; sleep-deprived patients exhibited a reduced synthesis of miR-182-5p, which resulted in the accumulation of ROS and proinflammatory events.

An early, formal, placebo-controlled clinical trial (NCT00640094) investigated the ability of melatonin to reduce morphophysiological damage to the human heart following the obstruction of a coronary artery [114]. The patients in this study experienced an ST-segment elevation myocardial infarction (STEMI). As soon as possible after diagnosis, the subjects were treated percutaneously with melatonin, immediately after balloon angioplasty of the occluded artery; melatonin was administered either intravenously or as an intracoronary bolus. A week following the cardiac procedure, magnetic resonance imaging (MRI) was used to evaluate the extent of cardiac damage. In the patients who received melatonin within an interval of up to 136 min after balloon opening and melatonin administration, assessed infarct size showed that it was reduced by roughly 40%. In subjects where the lag time after treatment was longer than 136 min, there was no statistically significant benefit as judged by MRI, and when melatonin was given more than 4 h after balloon placement, the lesion size tended to increase. The authors concluded that for melatonin to be useful as a treatment for STEMI, it should be given as soon as possible after the obstructed coronary artery is opened. This would seem to be consistent with what is known about reperfusion injury, where the influx of oxygenated blood quickly leads to oxidative stress and enlarged infarct volume; considering melatonin’s antioxidant effects, this is the interval where melatonin would be expected to provide its greatest protection. In consideration of these data, along with those of many other experimental studies illustrating the ability of melatonin to reduce heart damage after interruption of blood flow in a coronary artery, additional clinical trials are urgently needed [41,115,116].

Other limited trials that used melatonin to protect cardiac tissue from oxygen/glucose deprivation in humans have been performed using circulating troponin levels, left ventricular ejection fraction (LVEF), and MRI to assess infarct volume as indices of melatonin’s ability to reduce the severity of a heart attack [117,118,119,120]. The study paradigms differed among these investigations and, in some cases, it was uncertain whether a study was adequately blinded or whether the number of patients was large enough, and there was variability in the evaluated parameters. Of the measured outcomes, LVEF was found to be the most uniformly diminished and restored by melatonin administration. Circulating troponin levels were usually lower after melatonin administration, and MRI-assessed infarct volume was reduced. Although the findings are generally indicative of melatonin being a protective agent against cardiac ischemia/reperfusion injury in humans, the clinical significance of the collected data from these trials remains to be determined. What is needed is a better definition of a more optimal dose, as well as the route, time, and duration of administration. It is imperative, however, that these questions be answered, given the potentially high efficacy of melatonin in reducing CVD.

There is a great need for additional trials which examine the actions of melatonin on cardiovascular disease. There are limitations to the trials that have already been performed. Such trials must be of sufficient duration to differentiate between changes in the experimental groups; for cardiovascular diseases (with the exception of ischemia/reperfusion injury), this may be a significant length of time. The dose of melatonin that is used must be adequate to combat the massive damage that occurs. Thus, pharmacological doses of melatonin would likely be required to counteract the progression of such highly active pathological processes. Finally, non-invasive yet highly sensitive methods for determining outcomes are needed. The authors feel that melatonin, because of its extremely low toxicity and very high safety profile, along with its already-documented beneficial experimental actions, should be thoroughly exploited to determine its value as an agent to resist CVD, which are major causes of premature death throughout the world.

## 11. Guidelines for Establishing Melatonin Doses

The specific doses of melatonin that are used as treatment to reduce some aspects of cardiac pathophysiology have not, thus far, been mentioned. While this is obviously an issue of great importance, especially when considering the human-equivalent dose (HED), the translation of melatonin doses used in animals to doses for human use is not a straightforward calculation. While there are several allometric methods that can be used to estimate the human dose based on that used in animal studies, none of the methods are optimal, especially when comparing them based on disproportionate differences in body sizes only. Among animal species, the magnitude of specific actions to drugs often varies widely, so these functional variations must also be a consideration when determining appropriate human doses; these differences can relate to pharmacokinetic and pharmacodynamic variations, receptor density/sensitivity differences, complex mechanisms of action, differential toxicities, and drug interactions, among others. This problem is further compounded with regard to melatonin, since some of its actions are circadian-dependent and, as a consequence, the time of administration may be a critical factor in determining its efficacy [121].

Cardinali [122] recently thoroughly discussed the four common methods of dose calculation for the human, i.e., the basic formula method, fractional equation method, ratio and proportion method, and the dimensional analysis method, with each having its own limitations. The US Food and Drug Administration has published guidance on how to estimate safety doses in human drug trials to establish the HED [123]. Cardinali [122] used a modification of these recommendations to calculate a human melatonin dose for use in the treatment of osteoporosis; the value of a daily dose was identified to be in the range of 1.0–1.5 mg/kg body weight (100–150 mg daily). This may be impacted by the melatonin preparation that is used (fast release, slow release, sustained release, nano-encapsulated, etc.) and by the route of administration: oral, sublingual, intravenous, etc. There is general agreement that melatonin has a high safety margin and produces few adverse effects, which are usually subjective [124,125]. Using the calculated dose of 1.0–1.5 mg/kg body weight as a guideline seems appropriate for treatment of human cardiac pathologies as well, since the melatonin dose employed to treat osteoporosis in animals is generally similar to that used to combat cardiac pathophysiology in these same species.

## 12. Conclusions and Perspectives

Global health is greatly impacted by CVD. Frequently, cardiac disorders are secondary to atherosclerosis-mediated processes that predominantly cause dysfunction of endothelial cells lining the coronary arteries.

The endothelium is more than merely a barrier between the vascular lumen and the arterial intima. These cells regulate vascular tone, impact neoangiogenesis and, importantly, influence blood coagulation, which is a major contributor to cardiac damage since it can lead to occlusion of the coronary vessel that deprives the downstream cardiomyocytes of oxygen and glucose; this causes loss of the cardiomyocytes and their replacement with non-contractile elements that severely compromise heart physiology. Thus, endothelial damage in an early event in the complex processes that characterizes what is known as cardiovascular disease. This review briefly summarizes many of the pathophysiological events that contribute to the mechanical disruption of normal cardiac function and to heart failure, a major cause of premature death throughout the world.

While melatonin has a variety of functions by which it protects the heart from damage, its ability to neutralized destructive reactive oxygen species, many of which are free radicals, seems to be a primary intervening protective action at multiple levels of atherosclerotic plaque formation that can delay the subsequent processes which lead to several subtypes of cardiovascular disease. Considering the frequency and severity of CVD, the search for molecules that protect the heart from damage, especially that resulting from malfunction of the coronary endothelium, is intense. The available medications, rather than having been designed to prevent cardiac diseases, were developed to improve the hemodynamic features and other symptoms associated with the damaged heart. By comparison, melatonin, an endogenously synthesized molecule, the production of which is markedly diminished in the aged, seems to have the ability to delay/prevent the processes that are the forerunners of heart disease. The data suggest that the loss of melatonin levels in the latter stages of life is a contributing factor to generalized endothelial dysfunction and atherosclerosis development. The beneficial actions of melatonin seem to be especially manifest at the level of the mitochondria, an organelle that is the site of the generation of most reactive oxygen species, while also being targeted by melatonin. Similar to prescription medications, melatonin may also have utility in improving the quality of life of humans with CVD.

New discoveries are continually being made in terms of the beneficial actions of melatonin on cardiac pathophysiology. Although the experimental evidence is consistently positive regarding the ability of melatonin to defer atherosclerotic plaque formation, stabilize the plaques, and reduce cardiomyocyte damage resulting from obstruction of a coronary artery, as well as its actions in supporting individuals suffering from heart failure, this information has been used sparingly at the clinical level. The reader is reminded that the bulk of serious cases of atherosclerosis and the frequency of coronary artery occlusion occur most frequently in the elderly, a stage in life where endogenous melatonin production is often at its nadir. Considering the very high safety margin of melatonin and its virtual lack of toxicity, it would be responsible to test it as a general protector against CVD. Some clinical trials have used melatonin in the treatment of various diseases, with the duration of treatment ranging from a few days up to a year; similarly, the doses of melatonin that were used ranged from 3 mg daily to 1 g per day. Within the very wide dose range and treatment duration, melatonin’s side effects are minimal. When reported, these disturbances included a mild headache, dizziness, or sleepiness, with their frequency usually being no more than that resulting with placebo treatment [47,126]. Also, although attempts have been made to do so, no lethal dose for 50% of the animals (LD50) has been determined. Since melatonin is normally produced in the body (of all vertebrates, invertebrates, and plants), substantial toxicity would not be expected.
